# Plant-Derived Anti-Human Epidermal Growth Factor Receptor 2 Antibody Suppresses Trastuzumab-Resistant Breast Cancer with Enhanced Nanoscale Binding

**DOI:** 10.1021/acsnano.4c00360

**Published:** 2024-05-20

**Authors:** Chanyong Park, Kibum Kim, Yerin Kim, Rong Zhu, Lisa Hain, Hannah Seferovic, Min-Hyeok Kim, Hyun Joo Woo, Hyunju Hwang, Seung Ho Lee, Sangmin Kim, Jeong Eon Lee, Peter Hinterdorfer, Kisung Ko, Sungsu Park, Yoo Jin Oh

**Affiliations:** † School of Mechanical Engineering, Sungkyunkwan University, Suwon 16419, Korea; ‡ Department of Medicine, Medical Research Institute, College of Medicine, Chung-Ang University, Seoul 06974, Korea; § Department of Applied Experimental Biophysics, Institute of Biophysics, Johannes Kepler University Linz, 4040 Linz, Austria; ∥ Major of Nano-Bioengineering, College of Life Sciences and Bioengineering, Incheon National University, Incheon 22012, Korea; ⊥ Department of Breast Cancer Center, Samsung Medical Center, Sungkyunkwan University School of Medicine, Seoul 06351, Korea; # Division of Breast Surgery, Department of Surgery, Samsung Medical Center, Sungkyunkwan University School of Medicine, Seoul 06351, Korea

## Abstract

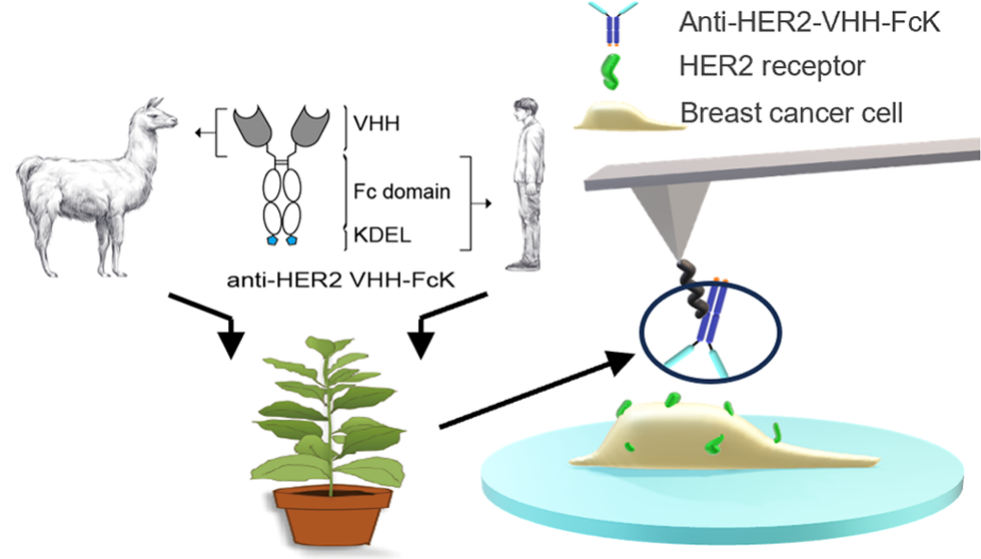

Traditional monoclonal antibodies such as Trastuzumab encounter limitations when treating Human Epidermal Growth Factor Receptor 2 (HER2)-positive breast cancer, particularly in cases that develop resistance. This study introduces plant-derived anti-HER2 variable fragments of camelid heavy chain domain (VHH) fragment crystallizable region (Fc) KEDL(K) antibody as a potent alternative for overcoming these limitations. A variety of biophysical techniques, *in vitro* assays, and *in vivo* experiments uncover the antibody’s nanoscale binding dynamics with transmembrane HER2 on living cells. Single-molecule force spectroscopy reveals the rapid formation of two robust bonds, exhibiting approximately 50 pN force resistance and bond lifetimes in the second range. The antibody demonstrates a specific affinity for HER2-positive breast cancer cells, including those that are Trastuzumab-resistant. Moreover, in immune-deficient mice, the plant-derived anti-HER2 VHH-FcK antibody exhibits superior antitumor activity, especially against tumors that are resistant to Trastuzumab. These findings underscore the plant-derived antibody’s potential as an impactful immunotherapeutic strategy for treating Trastuzumab-resistant HER2-positive breast cancer.

Breast cancer is a major health concern affecting more than 2 million women worldwide and its rising incidence and high mortality rates make it a major economic and social burden.^1−4^ Breast cancer can be classified into 4 molecular subtypes based on the presence or absence of estrogen receptor, progesterone receptors, and the human epidermal growth factor receptor 2 (HER2).^2,5−8^ Among these subtypes, breast cancer characterized by the overexpression of HER2 is linked to lower survival rates, presenting significant treatment challenges.^9,10^

Monoclonal antibodies (mAbs) such as Trastuzumab have been developed to inhibit the HER2 signaling pathway by binding to its extracellular domain.^5,11,12^ However, the efficacy of these mAbs is limited, with up to 60% of patients not responding to Trastuzumab.^1,2,13^ For example, several molecular variations within the HER2 protein hinder its binding to Trastuzumab, contributing to the treatment resistance. Therefore, this limitation has led researchers to explore alternative therapies that can penetrate tumors better, benefit resistant patients, and identify new binders’ affinity.^14,15^ One promising approach is the development of smaller antibody fragments that retain the intact antibody’s specificity and affinity. Variable fragments of camelid heavy chain domains (VHHs) are particularly suitable for targeting and internalizing antigens in poorly vascularized tissues such as tumors.^16,17^ VHHs have a lower molecular weight than full-size mAbs, enabling better tumor penetration. The smallest variable domain of the heavy chain, with a molecular weight of 15 kDa, possesses the ability to bind to antigens and is highly adaptable, facilitating the expression of diverse antibodies within a single cell.^18^ Moreover, this domain exhibits both physical and chemical stability, presents minimal immunogenicity, and serves as an exceptionally adaptable binding molecule. Despite these advantages, its production is constrained by the limitations of traditional heterologous systems for expressing therapeutic recombinant proteins, leading to high production costs that complicate large-scale manufacturing.^19^ Consequently, there is a pressing need for ongoing research aimed at devising more efficacious therapies and personalized treatment strategies for HER2-positive breast cancer.

To achieve cost-effective production of VHHs, researchers have explored the use of plants as an alternative host for heterologous protein expression.^20,21^ Plants offer several advantages over other expression systems, including safety from zoonotic diseases, low cultivation costs, glycosylation processes, and the potential for easy scalability through indoor and outdoor production.^1,20,22^ Among various methods to enhance the yield of therapeutic proteins in plants, achieving stable expression via nuclear transgenic plant techniques stands out as a particularly promising approach for large-scale manufacturing. This method enables stable gene insertion and simplifies the propagation and storage of transgenic plants via seeds.^23^ In our earlier investigation, we achieved the successful expression and purification of the anti-HER2 VHH-FcK antibody, which specifically targets breast cancer cells utilizing transgenic plants. This achievement was realized through fusion of the human immunoglobulin G (IgG) Fc domain and KDEL endoplasmic reticulum (ER) retention sequence to the VHH fragment.^1^ This antibody demonstrated its effectiveness in inhibiting the *in vitro* growth and migration of BT-474 cell line, which is characterized by HER2 positivity.^1^ However, it is important to highlight that our previous study did not include assays to evaluate the anti-HER2 activity of the VHH-FcK antibody against HER2-positive breast cancer cells that are resistant to Trastuzumab. Equally unexplored are the intricate binding mechanism and nanomechanical properties associated with the VHH-FcK antibody.

Over the past few decades, atomic force microscopy (AFM)-based force spectroscopy has emerged as a powerful tool for analyzing ligand binding strength and dynamics on living cell membranes at single-molecule resolution. Several studies have deciphered the binding mechanisms and inhibition processes of antibodies,^24,25^ peptides,^26^ cell adhesion molecules,^27^ and enzymes.^28^ Moreover, investigating the binding of mAbs to cancer cells and the subsequent activation of immune cells has provided insight into the mechanisms underlying cancer immunotherapy.^29^ Force spectroscopy has also pioneered the binding of single viral proteins and particles to host cells, deciphering the initial stages of viral infection^30−32^ and analyzing the molecular dynamics of lectin binding to pathogens aids the development of therapeutic strategies.^33,34^

Here, we study the anticancer mechanism of anti-HER2 VHH-FcK against wild-type (WT) HER2-positive breast cancer (BT-474) and its Trastuzumab-resistant cells (BT-474 TR). We decipher the nanomechanical kinetic and equilibrium binding mechanisms of the developed antibody with single-molecule resolution on living cells to obtain a detailed understanding of the antibody inhibition process. To this end, we use the AFM-based single-molecule force spectroscopy technique and deduce the interaction strength and kinetics during bimodal antibody bond formation and dissociation from HER2.

## Results

### Isolation and Characterization of Plant-Derived Anti-HER2 VHH-FcK Antibodies Targeting HER2

**Figure 1:**
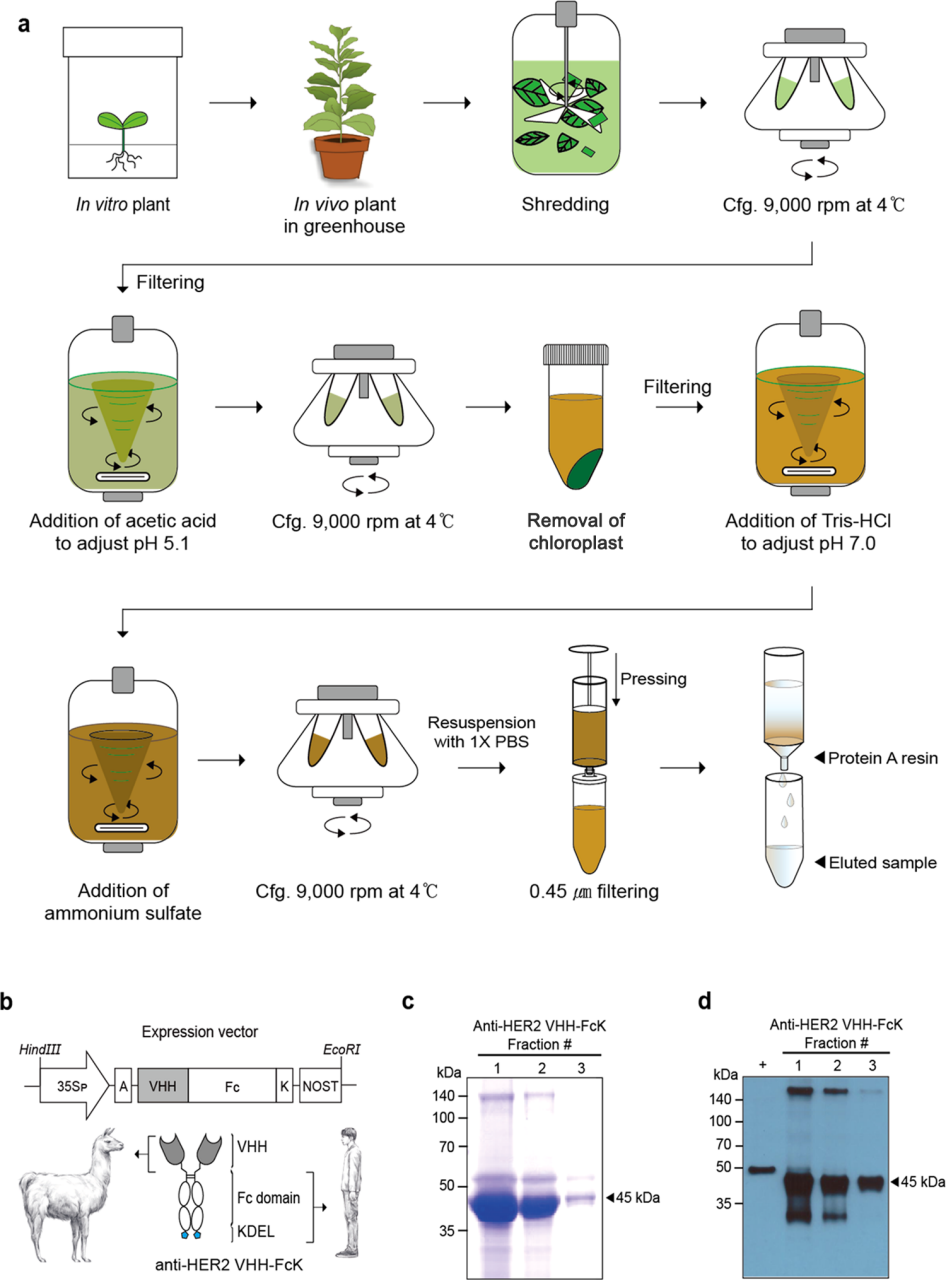
Expression and purification of the anti-HER2 VHH-FcK in transgenic tobacco plants. (a) Schematic diagram illustrating the purification of anti-HER2 VHH-FcK from transgenic plant leaf biomass.^35^*In vitro* transgenic plant seedlings carrying the anti-HER2 VHH-FcK transgene were transplanted to cultivate *in vivo* soil-grown transgenic plants in the greenhouse. After the removal of chloroplast and cell debris using centrifugation and filtering processes. (b) Anti-HER2 VHH-FcK expression cassette in the plant expression vector for *Agrobacterium*-mediated tobacco plant.^1^ Key elements include 35SP, Cauliflower mosaic virus 35S promoter with duplicated enhancer region; VHH, camelid anti-HER2 VHH; Fc, human IgG constant region fragment; K, KDEL; ER, retention signal; and NOS-T, terminator of Nopaline synthase gene. The KDEL ER retention signal is tagged to the C-terminus of the IgG Fc. The scheme shows the structure of the anti-HER2 VHH-FcK protein purified from the plant; gray section and white circle indicate llama-VHH and human-IgG Fc, respectively. (c) Coomassie blue stained SDS-PAGE visualizing fractions #1, 2, and 3 of the purified anti-HER2 VHH-FcK (45 kDa) from transgenic plant leaf. (d) Western blot confirming the fraction samples of the purified anti-HER2 VHH-FcK recombinant protein using horse radish peroxidase (HRP)-conjugated goat anti-human IgG Fc antibody. +, HC (50 kDa) of Trastuzumab (commercial anti-HER2) at 50 ng.

We initially transplanted *in vitro* seedlings of transgenic plants to the greenhouse to obtain plant biomass (Figure 1a). Subsequently, anti-HER2 VHH-FcK was purified from *in vivo* transgenic plant leaf biomass (Figure 1a) and sodium dodecyl sulfate polyacrylamide gel electrophoresis (SDS-PAGE) analysis validated the successful purification of anti-HER2 VHH-FcK using Protein A affinity chromatography (Figure 1a,c). The expression of the anti-HER2 VHH-FcK protein was confirmed by Western blot (Figure 1d). The camelid heavy chain (HC) bearing an antigen-binding site (VHH) was fused to the human-Fc fragment (hFc) generating the VHH-Fc fusion protein as a single domain (∼45 kDa), which is smaller than the HC of full-size antibody (∼50 kDa) (Figure 1b–d). Trastuzumab, with a total molecular weight of 150 kDa, is composed of two heavy chains (each approximately 50 kDa) and two light chains (each approximately 25 kDa). This structure is significantly larger than that of the anti-HER2 VHH-FcK, which has a molecular weight of around 90 kDa.

### Binding of Anti-HER2 VHH-FcK to HER2 on Breast Cancer Cell BT-474 and BT-474-TR Is Specific

**Figure 2:**
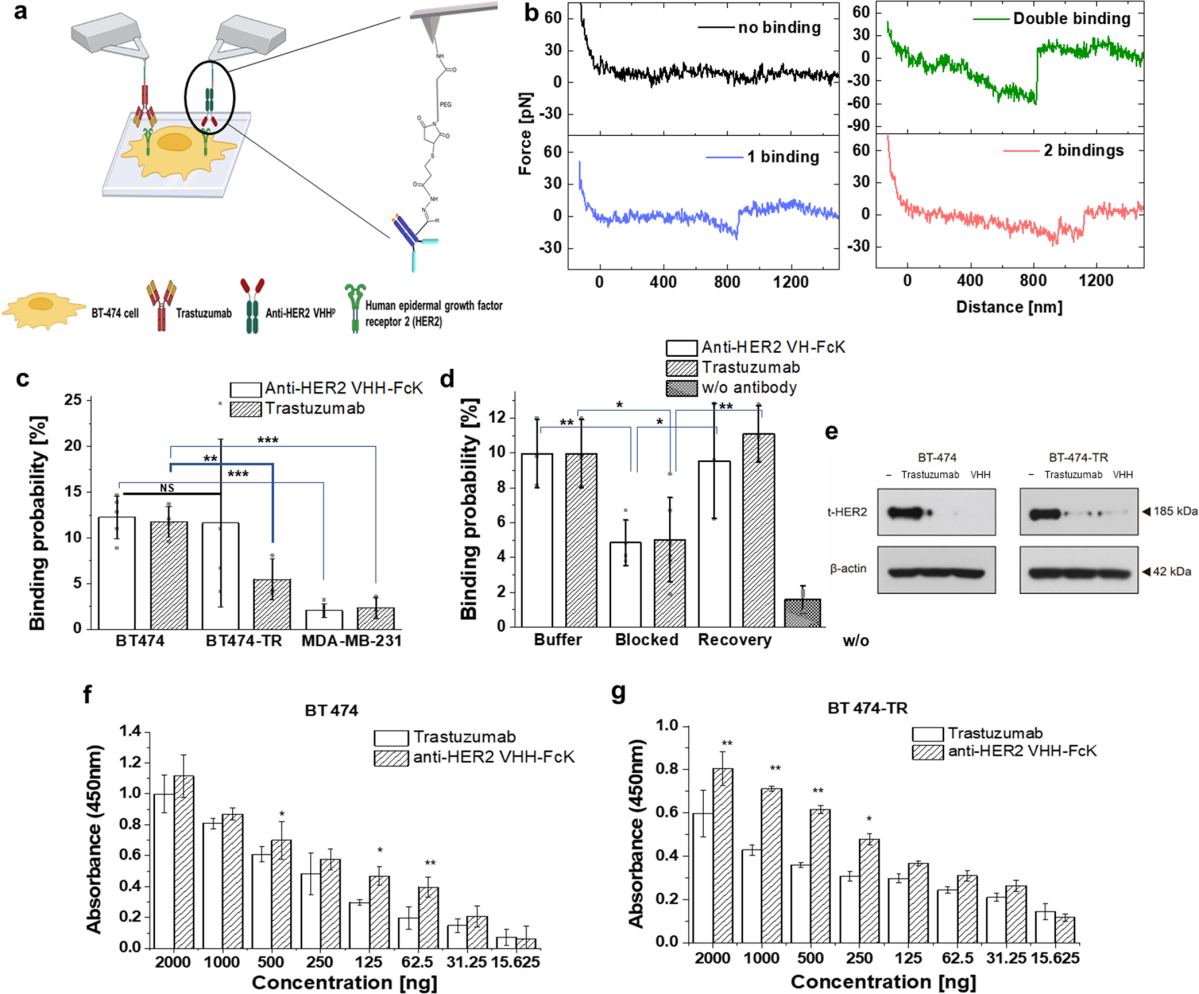
Single-molecule force spectroscopy (SMFS) measurements. (a) Schematic overview of the SMFS setup. Trastuzumab and anti-HER2 VHH-FcK were coupled to AFM cantilever tips for interaction studies with breast cancer cells expressing the HER2 receptor (inset: coupling of periodate-treated antibodies to AFM tip via maleimide-PEG27-NHS with stable hydrazone linkage). (b) Representative force traces showing typical bond ruptures (black: no binding, blue: single rupture, green: 2 simultaneous ruptures, red: 2 sequential ruptures). (c) Binding probabilities of anti-HER2 VHH-FcK and Trastuzumab with BT474 (*N* = 5), BT-474-TR (*N* = 4), and the triple-negative breast cancer cell line MDA-MB-231 (*N* = 3). (d) Binding probability of anti-HER2 VHH-FcK and Trastuzumab on BT474 surface before (*N* = 3), during blocking by injection of free anti-HER2 VHH-FcK or Trastuzumab into solution (*N* = 4), after washout (recovery (*N* = 3)), and of PEG linker without antibody on BT474 surface (*N* = 3). (e) Western blot of cell lysate treated with Trastuzumab and anti-HER2 VHH-FcK. BT-474 (left) and BT-474-TR (right). (f–g) Cell-based Enzyme-Linked Immunosorbent Assay (ELISA) was conducted to confirm the binding activity of Trastuzumab and anti-HER2 VHH-FcK to BT-474 (f) and BT-474-TR cells (g). Student’s *t* test; ***; *p* < 0.001, **; *p* < 0.01, *; *p* < 0.05, NS; No significant.

To decipher the detailed molecular mechanisms underlying the biochemical activity of Anti-HER2 VHH-FcK and Trastuzumab, we performed binding studies at the single-molecule level in HER2-positive breast cancer cells. The experimental principle of the approach is shown in Figure 2a. An antibody (Trastuzumab or anti-HER2 VHH-FcK) was coupled to the AFM tip using a hydrazone linkage method via a 6 nm long flexible poly(ethylene glycol) (PEG) cross-linker. By applying this specially tailored linker attachment to one of the small oligosaccharide chains (e.g., terminal glycan epitope mannose, GlcNAc) on the Fc arm of the antibody next to the hinge region (Figure 2a),^1,36^ we avoided random coupling to one lysine residue of the overall antibody structure or asymmetric orientation. This linkage also ensured sufficient motional freedom of the antibody for specific binding. Bringing an antibody-adorned AFM tip into contact with the surface of BT-474 leads to the formation of up to two cognate molecular bonds between the two Fab arms of the antibody and the HER2 molecules. Thereafter, the AFM cantilever was retracted to measure the unbinding force required to break the molecular bonds (Figure 2b). During retraction, an increasing force load was applied to the molecular bond, which was directly monitored from the downward deflection of the AFM cantilever. The loading force increased in a nonlinear parabolic-like fashion, characteristic for (i) the extension of the distensible PEG cross-linker (6 nm extended length) through which the antibody molecule is connected to the AFM tip and (ii) dominated from stretching the highly elastic cellular membrane (0 to 800 nm).^37^ Finally, at a critical force, termed the unbinding force, the antibody-linked tip detached from HER2 and the cantilever jumped back to its neutral position. In many of the recorded force–distance curves, either single-bond unbinding signatures (blue in Figure 2b) or double-bond signatures (green for simultaneous and red for sequential unbinding, in Figure 2b) were detected.

To confirm the specificity of the molecular bonds formed between the antibodies (anti-HER2 VHH-FcK/Trastuzumab) and HER2, we analyzed the binding probability, defined as the percentage of force measurements that displayed a specific unbinding event. In the case where a single curve in the analysis showed sequential binding behavior, only the last unbinding was considered and counted. Measurements were conducted on the two different cell surfaces, BT-474 and BT-474-TR (Figure 2c). Specific unbinding events were observed in similar frequency (about 13%) for both anti-HER2 VHH-FcK and Trastuzumab on BT-474. Importantly, anti-HER2 VHH-FcK also exhibited a significant binding probability (13%) on BT-474-TR, whereas Trastuzumab binding (5%) was largely absent. The specificity of the interactions of both anti-HER2 VHH-FcK and Trastuzumab was confirmed through independent control experiments conducted on the triple-negative breast cancer cell line MDA-MB-231, which does not express HER2 and BT-474 KD (HER2 knockdown BT-474, Figure S1) cells. These experiments demonstrated a significant decrease in the binding probability, with about 80% reduction observed on MDA-MB-231 cells and about 60% reduction on BT-474 KD cells (Figures 2c and S2). In additional block experiments (Figure 2d), injecting free anti-HER2 VHH-FcK antibodies or Trastuzumab into the measurement solution abolished the specific unbinding events due to the inactivation of the tip-adorned molecules. Washing the sample and tip with the measurement buffer led to recovery of the specific binding signals to about the same level. In measurements on BT-474 cells using AFM tips, lacking antibody binding was largely absent (Figure 2d). Conclusively, all control experiments unequivocally validated that the measured unbinding events detected with AFM tips carrying anti-HER2 VHH-FcK or Trastuzumab arose from specific molecular bindings of the antibodies to HER2.

The specificity of antibody binding was further validated in Western blots. Trastuzumab induces internalization and degradation of HER2 overexpressed on HER2-positive breast cancer cells.^38^ To analyze the potential of anti-HER2 VHH-FcK in HER2 protein degradation, Western blot analysis was performed using cells (BT-474 and BT-474-TR) treated with 20 μg/mL of anti-HER2 VHH-FcK and Trastuzumab as a control (Figure 2e). Anti-HER2 antibodies were used to detect HER2 protein in cell lysates. In BT-474 cells treated with Trastuzumab and anti-HER2 VHH-FcK antibodies, the density of the t-HER2 protein band was barely detectable (Figure 2e, left). Likewise, BT-474-TR cells treated with Trastuzumab and anti-HER2 VHH-FcK antibodies showed t-HER2 protein band densities significantly less pronounced than those of the negative control (Figure 2e, right). In both BT-474 and BT-474-TR cells, the cell lysate treated with the anti-HER2 VHH-FcK showed more degradation of t-HER2 protein compared to the cell lysate treated with Trastuzumab. Anti-HER2 VHH-FcK is therefore effective for both HER2-positive breast cancer cell lines, BT-474 and BT-474-TR.

In the cell ELISA, BT-474, its isogenic BT-474 KD and Mock (BT-474 cells transfected with vehicle alone), BT-474-TR, and HER2-negative colorectal cancer cells (SW480) were cultured in cell media. Subsequently, we conducted cell ELISA detection to analyze the binding affinity of plant-derived anti-HER2 VHH-FcK and Trastuzumab to the cell lines (Figures 2f,g, S3, and S4). When anti-HER2 VHH-FcK or Trastuzumab was introduced into BT-474 cells (Figure 2f), the absorbance decreased similarly in proportion to the concentration. Intriguingly, for BT-474-TR cells, the binding of anti-HER2 VHH-FcK was significantly more pronounced compared with that of Trastuzumab (Figure 2g).

In SW480 cells, neither Trastuzumab nor anti-HER2 VHH-FcK showed any detection signals across all concentrations (Figure S3). In all of the BT-474 cells (Figure S4), the absorbance levels of anti-HER2 VHH-FcK were higher than those of Trastuzumab. In BT-474 KD, both Trastuzumab and anti-HER2 VHH-FcK showed lower signals across all concentrations compared with BT-474 WT and BT-474 Mock cells. For BT-474 WT, BT-474 KD, and BT-474 Mock cells, the absorbance levels of anti-HER2 VHH-FcK were significantly higher when compared with Trastuzumab. These results indicate that anti-HER2 VHH-FcK has higher binding activity to HER2 cells when compared to Trastuzumab, with the most distinct effect on BT-474-TR cells.

### Dynamics of the Anti-HER2 VHH-FcK/HER2 Interactions

**Figure 3:**
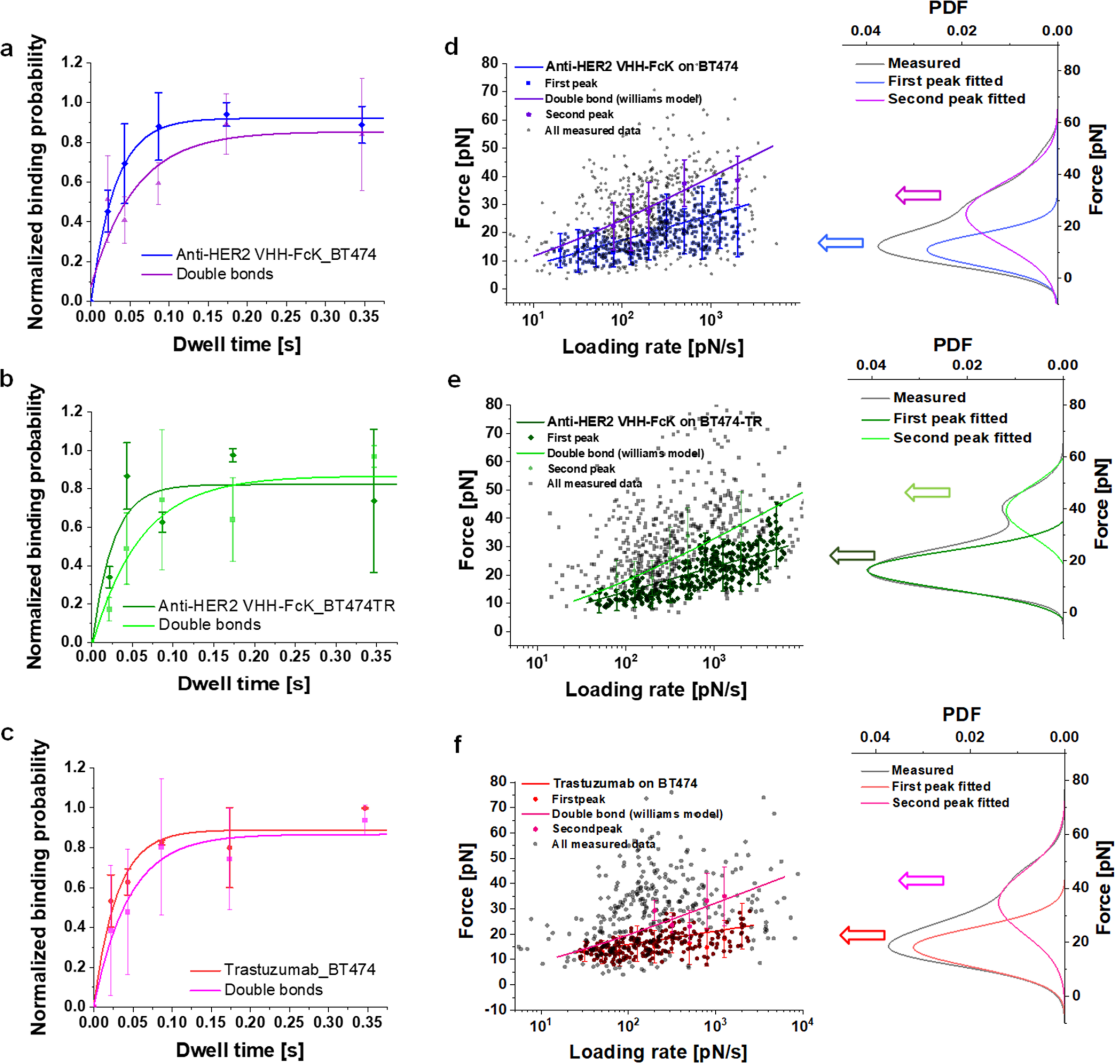
Molecular single- and multibond analysis of anti-HER2 VHH-FcK and Trastuzumab. (a–c) Dependence of the probability for binding and double-bond formation between antibody and cell as a function of the dwell time between (a) anti-HER2 VHH-FcK and BT474, (b) anti-HER2 VHH-FcK and BT-474-TR, and (c) Trastuzumab and BT474. (d–f) Plots of individual unbinding forces versus their loading rate for antibody dissociation from the cell surface (left) and examples of experimental probability density functions (PDFs) of unbinding forces from confined loading rate ranges fitted with the sum of two Gaussians (right). (d) Anti-HER2 VHH-FcK/BT474, (e) Anti-HER2 VHH-FcK/BT-474-TR, and (f) Trastuzumab/BT474 cell surface. Unbinding forces from single-bond ruptures (dark-colored dots) were fitted using the Evan’s single-barrier model (dark-colored lines). A Markov binding model, using parameters from the Evan’s fit computed (light-colored lines) the behavior of the unbinding forces of double bonds (light-colored dots).

We then derived the kinetic on-rate constant (*K*_on_) of antibody binding, for which we approximated the dependence of the binding probability (i.e., the probability to observe an unbinding event in a force–distance curve or not) on the tip-cell dwell time computing a pseudo-first-order kinetic eq (Figure 3a–c). More precisely, by varying the vertical scanning speed in the force–distance measurements, we set times in which the AFM tip was in contact with the cellular surface (denoted as dwell time) to eventually enable antibody/HER2 bond formation. Dwell times were calculated by determining the contact region of the force–distance curves (from 0 to about −150 nm, see slope on the left part of the force–distance curves in Figure 2b) for both the approaching and retracting part, divided by the vertical scanning speed of the tip. Prolonging the dwell time of the antibody-functionalized tip on the cell surface led to a significant increase in the binding probability that finally saturated (Figure 3a–c). We performed a least-squares fit to the data shown in Figure 3a–c and computed them according to
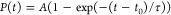
1where *t*_0_ represents the lag time and *A* represents the saturated binding probability. *K*_on_ was calculated using
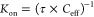
2with *C*_eff_ being the effective concentration of antibodies coupled to the AFM tip.^27,32^*C*_eff_ is given by *C*_eff_*=* 1/(*A*_C_*V)*, where *A*_C_ is the Avogadro constant and *V* is the effective volume of a hemisphere with radius *r*_eff_,^32^ over which the tip-adorned antibody can freely move. The effective radius was estimated as the sum of the cross-linker length (∼6 nm) in equilibrium and the half-length of antibody (∼4 nm; antibody is linked on the Fc arm close to the hinge region, using a hydrazone linkage method).^36^ Given that only one single antibody has access to the receptors on the cellular surface under our experimental conditions, *C*_eff_ is the reciprocal of the effective volume *V*. The *K*_on_ values (Table 1) for the association of Trastuzumab and anti-HER2 VHH-FcK to BT-474 cells were comparable (5.96 × 10^4^ and 4.79 × 10^4^ M^–1^ s^–1^, respectively), whereas Trastuzumab did not bind at all to the resistant BT-474-TR cells (Figure 2c) and anti-HER2 VHH-FcK preserved good binding activity (*K*_on_ = 3.60 × 10^4^ M^–1^ s^–1^). We also analyzed the time course for formation of the second antibody bond over time and computed the probability for double-bond formation as a function of the dwell time (pseudo-first-order kinetic equation, Figure 3a–c). The kinetic rates for double-bond formation, *K*_on_2bonds_, for the nanobody anti-HER2 VHH-FcK (*K*_on_2bonds_ ∼ 17 s^–1^) was somewhat smaller than for the antibody Trastuzumab (*K*_on_2bonds_ ∼ 22 s^–1^).

**Table 1:** Quantification of Parameters Obtained with the Dynamic Force Spectroscopy Method^a^

	anti-HER2 VHH-FcK		Trastuzumab
	BT-474	BT-474-TR	BT-474
*K*_off_ [s^–1^]	2.3 × 10^–1±0.6^	7.7 × 10^–1±1.5^	3.1 × 10^–1±2.8^
*K*_off_2bonds_ [s^–1^]	1.5 × 10^–1±1.0^	5.1 × 10^–1±4.0^	2.1 × 10^–1±1.0^
*K*_on_ [M^–1^ s^–1^]	4.79 × 10^4±2.07^	3.60 × 10^4±0.23^	5.96 × 10^4±2.33^
*K*_on_2bonds_ [s^–1^]	1.78 × 10^1±0.73^	1.67 × 10^1±0.62^	2.22 × 10^1±0.49^
*K*_D_ [M]	4.80 × 10^–6±3.9^	2.13 × 10^–5±0.1^	5.20 × 10^–6±3.3^
*X*_β_ [Å]	12.3 ± 1.57	11.05 ± 0.49	14.01 ± 6.46

a Data represent mean and errors from at least three independent measurements. Errors in the kinetic rates and *K*_D_ values are expressed in the exponent as logarithmic errors, as fits contain normal errors on log (*k*), and not on *k*.

To attain the dissociation rate constant (*K*_off_) of the cellular interactions of anti-HER2 VHH-FcK and Trastuzumab, we varied the pulling speed over a wide range. Individual unbinding forces were plotted versus their force loading rates (Figure 3d–f, left). There, the force loading rate of each individual force measurement was calculated by multiplying the retraction velocity with the effective spring constant from two springs in parallel, the spring constant of the cantilever and that of the molecules at the point of bond dissociation in force–distance curves. The binding valency of anti-HER2 VHH-FcK and Trastuzumab was retrieved in its distribution of unbinding forces at a fixed retraction velocity. For the construction of experimental probability density functions (PDFs), measured unbinding forces (retraction velocity of 3000 nm/s) were first normalized to Gaussians of unitary area with their width containing the measurement error, given by the thermal fluctuation of the AFM cantilever after unbinding occurred (typically a few pN). Thereafter, the Gaussians were gathered and summed up to compute the PDFs. PDFs contain the original data and can be viewed as the equivalent of continuous force histogram, with their maxima representing the most probable unbinding force. PDFs of unbinding forces showed a bimodal distribution of unbinding forces, arising from the bond breakage of one or two Fab arms, and were fitted with the sum of two Gaussians (Figure 3d–f, right). We then took unbinding forces within the range of the maxima ±2σ of the first Gaussians (Figure 3d–f, left) to consider the single-bond characteristics and fitted the individual force vs loading rate pairs (dark-colored data points) using Bell′s single energy barrier model.^39,40^ According to this theory that a single energy barrier is crossed in the thermally activated regime, we observed a linear rise of the unbinding force with respect to a logarithmically increasing loading rate, as expected. The unbinding force *F* is given as a function of the loading rate *r*,
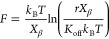
3where *k*_B_*T* is the Boltzmann thermal energy, *K*_off_ is the kinetic off-rate constant extrapolated to zero-force, and *X*_β_ is the length of the force-driven dissociation path for binding. In particular, a maximum likelihood approach was employed for fitting this statistical model with our data to retrieve the parameters of the model. The variance in the data in this plot mainly reflects the stochastic nature of the unbinding process. In addition to the most likelihood fit performed on multiple independent data sets for estimating the error for retrieving the kinetic off-rate constant (*K*_off_), mean values (dark-colored dots) and standard deviations, containing both measurement uncertainty and stochastic variation (dark-colored error bars), of the PDFs were added to the data fit. The *K*_off_ values for single-bond dissociation of anti-HER2 VHH-FcK and Trastuzumab from BT-474 yielded 2.3 × 10^–1±0.6^ s^–1^ and 3.1 × 10^–1±2.8^ s^–1^, respectively. Dissociation of one anti-HER2 VHH-FcK Fab arm from BT-474-TR was less resistant (*K*_off_ = 7.7 × 10^–1±1.5^ s^–1^).

Based on the fitting parameters of Evans’ theory, we used a Markov binding model^41^ to calculate theoretical forces for the dissociation of antibody double bonds from the two Fab arms (Figure 3d–f, left). The uncorrelated dissociation underlying this model implies that there is no mechanical coupling between the bonds. An excellent match of the data from the mean (light-colored dots) and standard deviation of the second Gaussian (light-colored error bars) from the higher force regime revealed independent dissociation of the two Fab arms of the antibodies from the cells. According to this model, the reduction of the overall kinetic off-rates arising from the rupture of two bonds, *K*_off_2bonds_, which characterizes dissociation of the whole antibody molecule, can be easily calculated from *K*_off_, using *K*_off_2bonds_ = *K*_off_/1.5. In agreement with our data, we derived *K*_off_2bonds_ for simultaneous 2 Fab unbinding of 0.15 s^–1^ for anti-HER2 VHH-FcK/BT-474, 0.51 s^–1^ for anti-HER2 VHH-FcK/BT-474-TR, and 0.21 s^–1^ for Trastuzumab/BT-474. Conclusively, the equilibrium dissociation constants *K*_D_ for the interactions of the whole antibodies were calculated from the ratio between *K*_off_2bonds_ and *K*_on_, yielding *K*_D_ ∼ 5.2 μM for Trastuzumab and *K*_D_ ∼ 4.8 μM for anti-HER2 VHH-FcK for binding to the BT-474. Binding of anti-HER2 VHH-FcK to the resistant BT-474-TR occurred with a lower affinity (*K*_D_ ∼ 21.3 μM).

### Migratory Response and Cytotoxicity of Breast Cancer Cell to Anti-HER2 VHH-FcK

**Figure 4:**
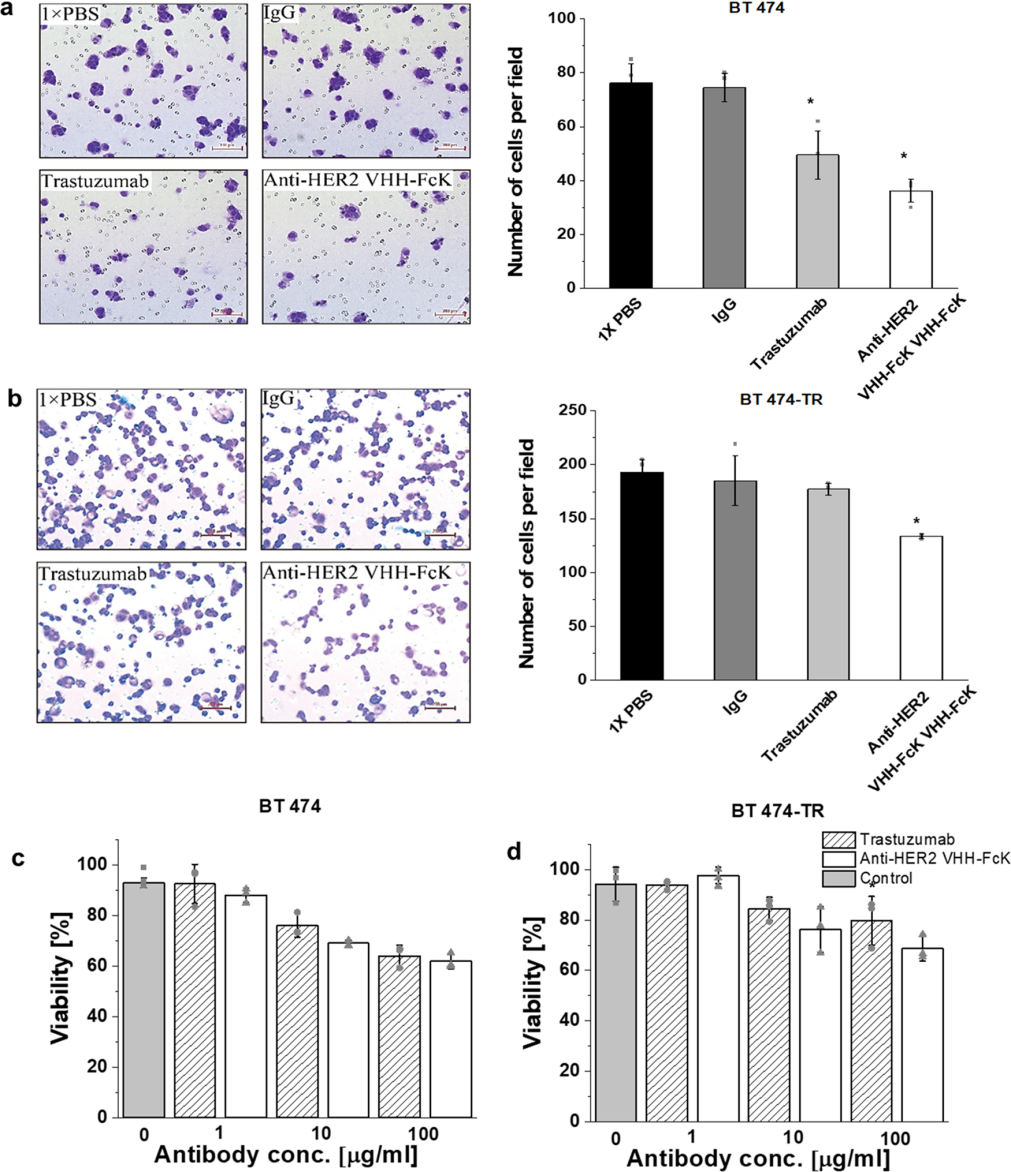
Transwell cell migration assay and morphologies of BT-474 and BT-474-TR cells treated with anti-HER2 VHH-FcK and Trastuzumab. (a, b) BT-474 (a) and BT-474-TR (b) cells were treated with 1× PBS, nonspecific IgG antibody as a negative control, Trastuzumab as a positive control, and anti-HER2 VHH-FcK. Left: optical microscopy images of migrated cells stained with 5% crystal violet; right: quantification of the number of cells per field and the comparison of antibody effects (anti-HER2 VHH-FcK and Trastuzumab) on inhibiting cell migration (* *p* < 0.05). (c, d) MTT assay for analyzing relative cell viability and *in vitro* sensitivity. BT-474 (c) and BT-474-TR (d) cells were treated with Trastuzumab and anti-HER2 VHH-FcK for 48 h at 3-fold dilutions (1, 10, and 100 μg/mL). Data points represent the average of three independent experiments with standard deviations indicated by error bars (* *p* < 0.05).

Cell migration inhibition was assessed using a transwell assay in which BT-474 and BT-474-TR cells were treated with anti-HER2 VHH-FcK and Trastuzumab, respectively (Figure 4a,b). The level of inhibition was determined by quantifying the number of crystal violet-stained cells that migrated to the bottom chamber through a defined collagen layer. The migrated cells were visualized using an optical microscope (Figure 4a,b, left), and the number of migrated cells per field was plotted (Figure 4a,b, right). For BT-474 cell migration (Figure 4a), the highest inhibition was observed when the cells were treated with anti-HER2 VHH-FcK (36.25 migrated cells), followed by Trastuzumab (49.5 migrated cells), the nonspecific IgG antibody (74.5 migrated cells), and the 1 × PBS-treated group (76.25 migrated cells). For BT-474-TR cells (Figure 4b), treatment with anti-HER2 VHH-FcK (36.25 migrated cells) resulted in a similar level of inhibition compared to BT-474, while Trastuzumab (54.75 migrated cells) had a less pronounced effect. The controls with the nonspecific IgG antibody (54.25 migrated cells) and the 1× PBS-treated group (60 migrated cells) yielded the expected results.

We then conducted a 3-(4,5)-dimethylthiazol-2-yl-2,5-diphenyltetrazolium bromide (MTT) assay to evaluate the effect of plant-derived anti-HER2 VHH-FcK on the viability of HER2-positive breast cancer cells (Figure 4c,d). The BT-474 and BT-474-TR cell lines, each seeded at a density of 5 × 10^3^ cells per well, were exposed to antibodies for a duration of 48 h. Subsequently, cell viability was assessed by measuring light absorbance at a wavelength of 550 nm utilizing an MTT assay kit. In both BT-474 and BT-474-TR cells, anti-HER2 VHH-FcK showed considerably lower viability compared with Trastuzumab across a range of concentrations. The results obtained from the MTT assay, conducted with antibody treatment in the absence of immune cell, indicate that anti-HER2 VHH-FcK has inherent cytotoxicity against HER2-positive breast cancer including Trastuzumab-resistant HER2-positive breast cancer. The reduced viability noted in both BT474 and BT-474-TR cell lines upon treatment with anti-HER2 VHH-FcK relative to Trastuzumab suggests that the anti-HER2 VHH-FcK exhibits superior cytotoxicity against cancer cells.

### Binding Activity of Anti-HER2 VHH-FcK to Fc Gamma Receptor (FcγRIIIa, CD16a)

**Figure 5:**
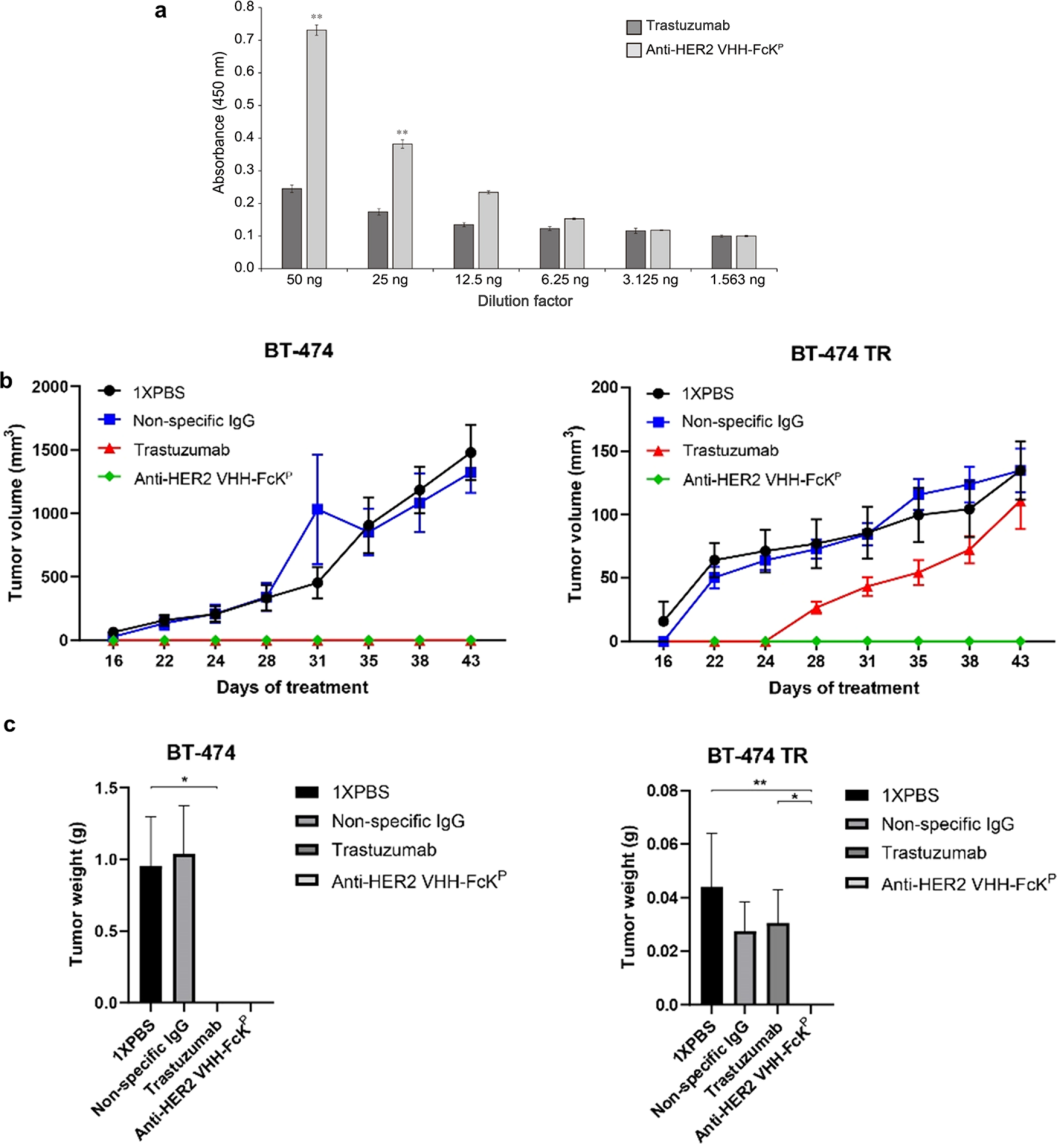
Binding activity of anti-HER2 VHH-FcK and Trastuzumab to Fc gamma receptor CD16a (FcγRIIIa), and tumor growth inhibition in NOD/Shi-scid/IL-2Rγnull mice bearing BT-474 and BT-474-TR. (a) ELISA analysis of anti-HER2 VHH-FcK and Trastuzumab binding to CD16a. CD16a was serially diluted 6-fold (** *p* < 0.01). (b) *In vivo* efficacy of anti-HER2 VHH-FcK and Trastuzumab for inhibition of tumor growth in NOD/Shi-scid/IL-2Rγnull mice for 43 days after BT-474 and BT-474-TR cell xenografting. (c) Tumor weight of BT-474 and BT-474-TR xenografted in mice 43 days after injection of anti-HER2 VHH-FcK and Trastuzumab. Tumor volume and size are expressed as mean ± SD (*n* = 5). Comparison between anti-HER2 VHH-FcK^P^ and Trastuzumab was performed using unpaired Student’s *t* test (Minitab software, Minitab, State College, PA). Statistical significance was denoted as follows: * *p* < 0.05, ** *p* < 0.01.

ELISA was performed to verify the binding activity of the Fc region to the CD16a known as FcγRIIIa, expressed on mast cells, macrophages, and natural killer cells, which activates antibody-dependent cellular cytotoxicity (ADCC),^42^ eventually leading to the elimination of primary leukemic cells, cancer cell lines, and cells infected with hepatitis B virus.^43^ In this study, we determined the binding affinity of anti-HER2 VHH-FcK to CD16a, one of the Fc gamma receptors (FcγRIIIa), and compared it with that of Trastuzumab, using ELISA analysis (Figure 5a). In detail, the surface of a 96-well plate was coated with FcγRIIIa (CD16a), and subsequently, anti-HER2 VHH-FcK and Trastuzumab were applied at serial dilutions. The binding interactions were then detected by measuring the HRP signal using an anti-His tag antibody and monitoring the absorbance at 450 nm (Figure 5a). The results showed that anti-HER2 VHH-FcK exhibited significantly higher binding affinity to CD16a when compared with Trastuzumab (Figure 5a).

### Tumor Inhibition Effect of Anti-HER2 VHH-FcK in BT-474 and BT-474-TR Xenograft Models

The activity of anti-HER2 VHH-FcK to bind and degrade HER2 protein on the surface of BT-474 and BT-474-TR cells *in vitro* as well as its inhibitory effect on cell proliferation, suggests the potential for its *in vivo* therapeutic efficacy. To assess this potential, we investigated the *in vivo* tumor growth inhibition of anti-HER2 VHH-FcK in HER2-positive breast cancer cell lines BT-474 and BT-474-TR. We used the NOG mice xenografted with BT-474 and BT-474-TR cells and administered treatments with anti-HER VHH-FcK and Trastuzumab. The tumor growth (0 mm^3^ and 0 g of tumor) was effectively suppressed in all mice when treated with BT-474 using anti-HER2 VHH-FcK and Trastuzumab (Figure 5b, left graph). In contrast, the control group of mice administered with 1× PBS and nonspecific IgG antibody continued to increase throughout the duration of the 43-day experiment. In mice with BT-474TR, tumors began to emerge on day 27 in mice treated with Trastuzumab (Figure 5b, right graph). Furthermore, tumors were observed as early as day 21 in mice subjected to the negative controls (1× PBS and nonspecific IgG antibody) and continued to grow until the end of the experiment (43 days). Notably, in mice treated with Trastuzumab, the final tumor volume and size were 110 mm^3^ and 0.03 g after 43 days of growth (Figure 5c, right graph). However, the tumor growth pattern in mice bearing BT-474-TR xenografts exhibited notable differences. While the tumor growth was effectively inhibited in all mice treated with anti-HER2 VHH-FcK, no such inhibition was observed with Trastuzumab treatment (Figure 5b, right graph). As anticipated, the control group of BT-474-TR xenografted mice administered with 1× PBS and nonspecific IgG antibody showed continuous tumor growth until the end of the 43-day experiment. In BT474 xenografted mice treated with 1× PBS and nonspecific IgG antibodies, the extracted tumors yielded average weights of 0.95 and 1.04 g, respectively (Figure 5c, left graph). Conversely, in BT474 xenografted mice treated with anti-HER2 VHH-FcK and Trastuzumab, the tumor weighed 0 g (Figure 5c, left graph). Similarly, for BT-474-TR xenografted mice administered with 1× PBS and nonspecific IgG antibodies, the extracted tumors had average weights of 0.43 and 0.023 g, respectively (Figure 5c, right graph). In contrast, BT-474-TR xenografted mice treated with anti-HER2 VHH-FcK exhibited no tumor growth, while those treated with Trastuzumab had tumors with a weight of 0.029 g (Figure 5c, right graph).

## Discussion

In this study, we monitored the antitumor activity of anti-HER2 VHH-FcK on Trastuzumab-resistant breast cancer cell BT-474-TR for potent anticancer activity. In addition, we deciphered the nanomechanical binding kinetics and energetics to anti-HER2 VHH-FcK binding to HER2 antigen expressed on human breast cancer cells BT-474 and BT-474-TR and compared it with that of Trastuzumab. At the single-molecule level using SMFS, we observed that anti-HER2 VHH-FcK binds specifically to HER2 on BT-474 as well as on BT-474-TR, whereas Trastuzumab shows particularly weak binding on the resistant cell surface. We obtained an affinity *K*_D_ of anti-HER2 VHH-FcK binding in the micromolar range on the surface of living cells in a physiological setting. The bimodal force distribution indicates that anti-HER2 VHH-FcK formed double bonds, likely via its 2 Fab fragments, with a bond lifetime of τ = 4.3 s (derived from *K*_off_ according to τ = 1/*K*_off_) on the HER2-positive tumor cells BT-474. This bond lifetime is longer than that observed for Trastuzumab (3.2 s) and in agreement with our ELISA assay (Figure 2f). The stronger adhesion of anti-HER2 VHH-FcK suggests greater antitumor potential compared to Trastuzumab. Additionally, anti-HER2 VHH-FcK demonstrates high specificity for HER2, even on the surface of Trastuzumab-resistant tumor cells, indicating its ability to target resistant tumors. Moreover, modulated kinetic binding constants (Table 1) suggest binding to other epitopes of HER2, expanding its potential therapeutic efficacy.

Binding of the anti-HER2 VHH-FcK antibody to an epitope distinct from Trastuzumab may enable it to more readily interact with HER2, even in situations in which the receptor is partially masked by glycoproteins. Preclinical studies have suggested that membrane proteins interacting with HER2, such as MUC4, can hinder the accessibility of trastuzumab to its epitope.^44^ MUC4, an overexpressed transmembrane glycoprotein in some breast tumors, interacts with and activates HER2, thereby impeding the binding of Trastuzumab to HER2. Our results suggest that by targeting a different epitope on HER2, the anti-HER2 VHH-FcK antibody may bypass the masking effect caused by proteins like MUC4 and exhibit improved binding efficiency.

Antibodies are widely used as targeted therapeutics because of their high specificity and affinity. However, they are often hindered by their large size, which limits their efficient diffusion through the tumor tissue. Additionally, they can be affected by the “binding site barrier” effect, attributed to their high affinity,^45,46^ so that the moderate micromolar binding affinity of anti-HER2 VHH-FcK might make it more effective. Bivalent binding of anti-HER2 VHH-FcK plays an important role in antibody inhibition, as evidenced by the bimodal force distributions recorded on the cell surfaces. Fast association (Table 1), combined with its smaller size compared to the Trastuzumab, may result in better conformational accessibility to the HER2 binding site on the surface of Trastuzumab-resistant cells. This alteration in binding dynamics between anti-HER2 VHH-FcK and HER2 on the Trastuzumab-resistant cell surface could ultimately lead to effective inhibition of HER2/HER2 or HER2/HER3 dimerization. The *in vitro* high affinity of anti-HER2 VHH-FcK for tumor cells, comparable to Trastuzumab, suggests it as a promising alternative therapeutic antibody for HER2-positive breast cancer.

In the cell migration assay, it is noteworthy that the numbers of migratory cells for both BT-474 and BT-474-TR, when treated with anti-HER2 VHH-FcK, were significantly lower than those treated with Trastuzumab as a positive control. Cancer cell migration is a pivotal factor in the process of metastasis, which should be inhibited to suppress the spread of cancer. Thus, these findings highlight the potential of anti-HER2 VHH-FcK as a promising therapeutic alternative for trastuzumab-resistant breast cancer.

In the experiment using immune-deficient mice, both anti-HER2 VHH-FcK and Trastuzumab effectively inhibited the growth of BT-474 tumors. Furthermore, anti-HER2 VHH-FcK additionally inhibited the growth of BT474-TR tumors, while Trastuzumab did not. It is important to note that the NOG (NOD/Shi-sci/IL-2^rnull^) mice utilized in this study are immunodeficient mice derived from NOD/SCID mice, featuring a common gamma chain mutation.^47^ These mice were developed through backcrossing C57BL/6J-IJ-2R mice deficient in the IL-2 receptor gamma chain with NOD/Shi-scid mice.^48^ The NOG mice have multifunctional defects in NK cell activity, macrophage function, complement activity, and dendritic cell function without functional T and B lymphocytes.^47,48^ Hence, it is unlikely that the inhibition of BT474 and BT474TR was due to the combinatorial interaction of both the anti-HER2 VHH nanobody and immune cells such as NK cells, macrophages, and dendritic cells. Indeed, Trastuzumab, used as a positive control, has been found to block the interaction between HER2/HER-3 and to inhibit critical downstream signaling pathways, ultimately leading to the suppression of tumor cell growth.^47^ Furthermore, Trastuzumab has been reported to induce normalization and regression of the tumor vasculature.^49^ We speculate that the anti-HER2 VHH nanobody may block the homodimerization and heterodimerization of HER2/HER3, leading to a downregulation of HER2 receptor signaling and subsequent inhibition of cancer cell proliferation. This note is based on the similar bivalent binding capacity of the anti-HER2 VHH nanobody when compared with Trastuzumab, which has been reported to block the interaction between HER2/HER3 and inhibit downstream signaling pathways.^50^ The single domain of anti-HER2 VHH-Fc can be co-expressed with other targeting VHH antibodies in a single plant, eventually expressing bispecific or multispecific antibodies. Fc fusion to VHH could provide ADCC function to anti-HER2 VHH-Fc for anticancer activity. Our results suggest that the anti-HER2 VHH-FcK antibody could be used as a potential immunotherapeutic option for patients with Trastuzumab-resistant breast cancer.

## Conclusions

Our study represents a pioneering investigation comparing Trastuzumab with a plant-derived nanobody, anti-HER2 VHH. The advantages of plant-based expression systems, including cost-effectiveness, scalability, and safety, make them an attractive platform for producing various therapeutic antibodies. Through SMFS, we uncovered stronger adhesion of anti-HER2 VHH-FcK to Trastuzumab-resistant tumor cells compared to Trastuzumab. Furthermore, our findings revealed the high specificity of anti-HER2 VHH-FcK for HER2, even on the surface of Trastuzumab-resistant tumor cells, showcasing its potential to target resistant tumors. Additionally, modulated kinetic binding constants (see Table 1) indicated binding to other epitopes of HER2, thus expanding its therapeutic efficacy. The bivalent binding of anti-HER2 VHH-FcK was pivotal in antibody inhibition, as evidenced by the recorded bimodal force distributions on cell surfaces. Notably, the rapid association kinetics of anti-HER2 VHH-FcK, coupled with its smaller size compared with Trastuzumab, may lead to enhanced conformational accessibility. These results collectively highlight the contribution of our biophysical approach, leveraging both AFM and cell biology techniques, to elucidate the distinct mechanisms and potential advantages of plant-derived antibodies in cancer therapy.

## Methods

### Plant Vector Establishment, Transformation, and Expression of Plant-Derived Anti-HER2 VHH-FcK Protein

*Agrobacterium*-mediated plant transformation with the plant expression vector pBI anti-HER2 VHH-FcK (Figure 1a,d) was conducted to generate tobacco plants expressing anti-HER2 VHH-FcK.^1^*In vitro* transgenic plant seedlings carrying the anti-HER2 VHH-FcK transgene were transplanted to cultivate *in vivo* soil-grown transgenic plants in the greenhouse (Figure 1a). To purify the anti-HER2 VHH-FcK, 200 g of the transgenic plant leaves were homogenized with the extraction buffer (Figure 1a).^35^ After the removal of chloroplast and cell debris using centrifugation and filtering processes, the anti-HER2 VHH-FcK proteins were purified through Protein A affinity chromatography (Amicogen, Jinju-si, Korea). The SDS-PAGE gel loaded with the purified target protein was stained with Coomassie blue staining buffer, and then the gel was destained by destaining buffer (Figure 1b).^51^

### *In Vitro* Cultivation of Human HER2-Positive Breast Cancer Cell Line (BT-474) and BT-474-Trastuzumab-Resistant Cell Line (BT-474-TR)

Human BT-474 and BT-474-TR were obtained from the Korean Cell Line Bank, Seoul, Korea. The BT-474 cells were cultured in RPMI 1640 medium (Welgene, Gyeongsan, Korea) supplemented with 10% fetal bovine serum (Invitrogen, Waltham, MA), 100 IU/mL penicillin, and 100 μg/mL streptomycin. The HER2-positive cells were cultured at 37 °C in an atmosphere of 95% air and 5% CO_2_ in an incubator (Thermo Fisher Scientific, Waltham, MA). The culture medium was refreshed by replacing it every 3 days. The human BT-474 Trastuzumab-resistant cells (BT-474-TR) were cultured by the Samsung Medical Center in Seoul, Korea. BT-474-TR cells were maintained in RPMI 1640 media (Life Technologies, Rockville, MD), supplemented with 10% FBS (Hyclone, Logan, UT), 100 IU/mL penicillin, and 100 μg/mL streptomycin in a humidified atmosphere consisting of 95% air and 5% CO_2_ in a CO_2_ incubator (Thermo Fisher Scientific, Waltham, MA).

### Transfection with siRNA

Short interfering RNA (siRNA) oligonucleotide duplexes targeting the genes of HER2 were synthesized and annealed by IDT (Integrated DNA Technologies, Inc., San Diego, CA) as following sequences: 5′-CCUGUGCCCACUAUAAGGA-3′ (sense) and 5′-UCCUUAUAGUGGGCACAGG-3′ (antisense). AccuTarget Control siRNA (Bioneer, Daejeon, Korea) was used as a negative control. Transfections were performed using Lipofectamine RNAiMAX Reagent (Thermo Fisher Scientific, Waltham, MA) according to the manufacturer’s instructions. After 48 h, the transfection efficiency was checked using quantitative real-time PCR (qRT-PCR) and Western blot analysis.

### Cell ELISA

A cell-based ELISA was performed to evaluate the binding affinity of anti-HER2 VHH-FcKP and Trastuzumab toward BT474, its isogenic HER2 knockdown counterpart (BT-474 KD), BT474 cells transfected with the vehicle alone (BT474 Mock), and the colorectal cancer cell line SW480.^1,13,52^ Cells were plated at a density of 5 × 10^3^ cells per well in a 96-well plate. For fixation, cell culture medium was removed, and cells were treated with 100 μL of 4% paraformaldehyde (PFA) in 1× PBS for 20 min at room temperature. Following fixation, cells were washed three times with 1× PBS and then blocked using 1% bovine serum albumin (BSA) in 1× PBS as a blocking buffer for 1 h and 30 min at room temperature. Following three washes with 1× PBS, cells were incubated with serial dilutions of Trastuzumab (Biovision, Inc., Milpitas, CA) and anti-HER2 VHH-FcK (ranging from 2.0 to 0.015625 μg) in blocking buffer, serving as the primary antibody treatment. After another set of three 1× PBS washes, HRP-conjugated goat anti-human IgG Fc antibody (Jackson ImmunoResearch Laboratories, Inc., West Grove, PA; diluted in blocking buffer at a 1:8000 ratio) was added as the secondary antibody and incubated for 2 h at 37 °C. The wells were then washed with 1× PBS before the addition of 3,3′,5,5′-tetramethylbenzidine (TMB) substrate solution (Seracare Life Sciences, Milford, MA) to each well, allowing for a 3 min signal development. To stop the TMB reaction, TMB stop solution (Seracare Life Sciences, Milford, MA) was added. Absorbance at 450 nm for each well was measured using a Gen5 microplate reader with version 2.01 software (BioTek Instruments, Inc., Winooski, VT).

### Transwell Migration Assay

Cell migration assay was performed to analyze *in vitro* cell invasion using Transwell Permeable Supports with 3 μm-pore size (Corning Inc., Corning, NY). Briefly, BT-474 and BT-474-TR cells (5 × 10^4^ cells) were each seeded in an upper chamber of a 24-well plate coated with type I collagen solution (Sigma, St. Louis, MO) in 1× PBS (10 μg/mL) overnight at 4 °C. The under chamber was filled with 1 mL of RPMI 1640 media containing 20% of FBS. The seeded cells were treated with anti-HER2 VHH-FcK, Trastuzumab (positive control), nonspecific IgG antibody (negative control), and 1× PBS at a final concentration of 20 μg/mL and incubated for 24 h at 37 °C in a CO_2_ incubator. And the migrated cells to the bottom chamber were fixed and stained by using 5% crystal violet. The stained cells were counted under a microscope.

### Cell Viability Assay

Cell viability was measured by the 3-(4,5-dimethylthiazol-2-yl)-2,5-diphenyl-2,5-diphenyltetrazolium bromide (MTT) assay (Sigma-Aldrich, St. Louis, MO). The cells used in the MTT assay were BT-474 and BT-474-TR, and 5 × 10^3^ cells were grown in RPMI 1640 medium (Welgene) supplemented with 10% FBS in a 96-well plate (SPL Life Sciences Co., Seoul, Korea). The final volume of cells grown in microplates was 100 μL per well, and then, the cells were incubated at 37 °C in a CO_2_ incubator (Thermo Fisher Scientific, Waltham, MA). After the incubation, the cells were treated with Trastuzumab (Biovision, Inc., Milpitas, CA) as positive control, anti-HER2 VHH-FcK at concentrations of 100, 10, and 1 μg/mL, respectively, and 1× PBS as negative control incubated overnight in a 37 °C in a CO_2_ incubator.

### Western Blot to Confirm Total HER2 Protein Level in BT-474 and BT-474-TR Treated with Anti-HER2 VHH-FcK Using Cellular Protein

The BT-474 and BT-474-TR cells were seeded in 6-well plates and incubated for 24 h at 37 °C in a CO_2_ incubator. After incubation, the cells were treated with commercial Trastuzumab (Biovision, Inc.) as a positive control (20 μg/mL), anti-HER2 VHH-FcK (20 μg/mL), and no treatment as a negative control for 16 h at 37 °C in a CO_2_ incubator. Whole-cell lysates (BT-474, BT-474-TR) were harvested using PRO–PREPTM Protein Extraction Solution (Intron Biotechnology, Inc., Sungnam-si, Korea) and centrifuged at 13,200 rpm for 15 min. Western blot was performed using the harvested antibody-treated cell lysates of BT-474 and BT-474–TR treated with Trastuzumab and anti-HER2 VHH-FcK. Total HER2 protein (t-HER2, 185 kDa) in cell lysates was detected by anti-HER2 antibody conjugated to horse radish peroxidase (HRP) (1:5000). An anti-β-actin antibody was used to confirm that the same amount of cell lysate was used for the Western blot analysis. In a similar way, the knockdown (KD) efficiency of the BT474-KD and BT474-Mock cells was confirmed.

### Cell ELISA to Confirm the Binding Activity of Trastuzumab and Anti-HER2 VHH-FcK to HER2-Positive Breast Cancer Cells BT-474 and Trastuzumab-Resistant Mutant Cell Line BT-474-TR Cells

HER2-positive breast cancer cells BT-474 and Trastuzumab-resistant mutant cell line BT-474-TR cells were fixed on the surface of a 96-well cell culture plate using 4% PFA overnight at 4 °C. Trastuzumab and anti-HER2 VHH-FcK were used as primary antibodies to HER2-positive breast cancer cells. The secondary antibody was HRP-conjugated goat anti-human IgG Fc antibody.

### ELISA Assay to Confirm the Binding Activity of Anti-HER2 VHH-FcK to Fc Gamma Receptor (Fcγ Receptor, CD16a)

Enzyme-linked immunosorbent assay (ELISA) was performed to compare the binding affinity between anti-HER2 VHH-FcK, Trastuzumab, and CD16a (Sino Biological, Beijing, China), a type of Fc gamma receptor (FcγRIIIa).^1^ 50 ng per well of anti-HER2 VHH-FcK and Trastuzumab in 100 mM bicarbonate/carbonate coating buffer (pH 9.6) were coated on the surface of a 96-well Maxisorp plate (Thermo Fisher Scientific, Waltham, MA) for overnight at 4 °C.

### *In Vivo* Assay for Tumor Growth Inhibition

Female 4-week-old NOG (NOD/Shi-scid/IL-2Rγnull) mice (KLS Bio, Suwon, Korea) were inoculated s.c. with 2 × 10^6^ human HER2-positive breast cancer cells (BT-474 and BT-474-TR). Immediately after tumor cell inoculation, four groups of five mice each were injected i.p. with 5 mg/kg of anti-HER2 VHH-FcK, Trastuzumab (positive control), and nonspecific IgG antibody (negative control), respectively, including 1× PBS followed by the same injections given every day for a total of 4 days. Tumor volumes were calculated based on the three major diameters measured with graduated calipers and were recorded 12, 19, 26, 33, and 40 days after injection. The size of the tumor was measured after the size of the tumor reached 50 mm^3^, and the formula used for tumor size measurement is as follows: volume = length × width × width/2. All animal experiments were conducted in compliance with the guidelines approved by the Animal Experiment Ethics Committee in accordance with the Animal Protection Act (Law No. 16977) (Approval Number: KLSIACUC20221130-4-01, KLS Bio Officer Lee). Mice were killed by CO_2_ inhalation on day 40 after tumor observation. Statistical analysis with Student’s *t* test was performed to test for the different tumor volumes of each group by using Minitab software (Minitab, State College, PA).

### Conjugation of Antibody through Carbohydrate Residues to AFM Tip

MSCT cantilevers (Type C, nominal frequency 7 kHz, spring constant 0.01 N/m, Length 310 μm, Width 20 μm, Q-factor ∼1, Bruker, Camarillo, CA) were washed three times with chloroform and dried in a gentle nitrogen gas stream directly before further treatment. Before use, APTES was freshly distilled under a vacuum. A desiccator was flooded with argon gas to remove the air and moisture. Then, 30 mL of APTES and 10 mL of triethylamine were separately pipetted into the chamber, while the AFM tips were placed nearby on a clean inert surface. Following incubation, APTES and triethylamine were removed, and the desiccator was again flooded with argon gas for 5 min. The tips were then left inside for 2 days to allow for curing of the APTES coating. The APTES-functionalized AFM tips were immersed in 0.5 mL of a solution containing 1 mg/mL of maleimide-PEG27-NHS in chloroform, with 0.5% (v/v) content, for 2 h. Subsequently, a mixture of EDTA, HEPES, TCEP hydrochloride, and water was prepared and pipetted onto the tips, followed by a 2 h incubation period. Afterward, the tips were washed three times with water and dried by using a gentle stream of nitrogen.

Antibodies were dialyzed using a mini dialysis tube in sodium acetate at 4 °C for 2 h. The antibody solution was collected from the dialysis tube into a 0.5 mL tube. NaIO_4_ in water was then added to the antibody solution. The reaction mixture was kept in the dark at room temperature. The antibodies treated with NaIO_4_ were dialyzed two times in the buffer. Then, periodate-treated and dialyzed antibodies were coupled to cantilever tips containing PEG–PDP^36^ before they were washed and stored in a buffer.

### Single-Molecule Force Spectroscopy Measurement

All measurements were carried out at room temperature by using a PicoPlus 5500 AFM setup (Agilent Technologies, Santa Clara, CA) on living cells containing HBSS. The functionalized cantilever with a nominal spring constant of 0.01 N/m was moved downward to the cell surface and moved upward after the tip touched it. Spring constants (*K*_c_) of AFM cantilevers were determined using the equipartition theorem^53^ in an ambient environment and ranged from 0.007 to 0.012 N/m. The deflection sensitivity was calculated from the slope of the force–distance curves recorded on a bare silicon substrate. At least 1000 force–distance curves (2000 data points per curve) were recorded with a typical force limit of 30–100 pN and vertical sweep rates between 0.1 and 2 Hz at a *z*-range of typically 3000 nm, resulting in loading rates from 50 to 5000 pN/s. The loading rates were determined by multiplying the pulling velocity with the effective spring constants (*K*_eff_), which were obtained by the spring constant of the cantilever (*K*_c_) and the spring constant of the PEG linker (*K*_L_), according to *K*_eff_ = [1/*K*_c_ + 1/*K*_L_]. *K*_L_ was calculated by fitting the force–distance curves with the work-like chain model as described.^32^

To investigate the blocking of HER2 on the surface, blocking experiments were conducted using free Trastuzumab and anti-HER2 VHH-FcK antibodies in solution, respectively. 10 μL of Trastuzumab and anti-HER2 VHH-FcK was incubated in the cell culture chamber for 30 min to block HER2 on the surface. Subsequently, the binding probability of the AFM tip-adorned HER2 and specific antibodies was measured. Thereafter, antibodies were washed out three times by using HBSS buffer. Final measurements were performed to assess the recovery of binding probability.

### Data Analysis

Force–distance curves were analyzed using Matlab (MathWorks, Inc., Natick, MA) with an integrated in-house package, which provides tools for the statistical analysis of the whole set of data, as described before. Unbinding events were identified by local maximum analysis using a signal-to-noise threshold of 2.^54,55^ The binding probability was calculated as the fraction of curves showing unbinding events. For example, if 200 curves from 2000 measured curves show unbinding events, then the binding activity is 10%.

### Statistical Analysis

All values are presented as the mean ± SD. Comparison between anti-HER2 VHH-FcK^P^ and Trastuzumab were performed using unpaired Student’s *t* test. Statistical analyses were conducted using Minitab software (Minitab, State College, PA). Statistical significance was denoted as follows: * *p* < 0.05, ** *p* < 0.01, *** *p* < 0.001.

## Data Availability

All data and materials used in this study will be available upon reasonable request.

## Supporting Information Available

Confirmation of HER2 KD in BT474 cells (Figure S1); single-molecule force spectroscopy control measurement (Figure S2); and cell ELISA to confirm binding activity of anti-HER2 VHH-FcK and Trastuzumab to SW480, BT474 WT, BT474 KD, and BT474 Mock cells (Figures S3 and S4) (PDF)

## Abbreviations

HER2: human epidermal growth factor receptor 2
mAbs: monoclonal antibodies
VHHs: variable fragments of camelid heavy chain domains
IgG: immunoglobulin G
ER: endoplasmic reticulum
AFM: atomic force microscopy
WT: wild type
PEG: poly(ethylene glycol)
ADCC: antibody-dependent cellular cytotoxicity
TMB: tetramethylbenzidine
