# Supplementary material for: Plant-Derived Anti-Human Epidermal Growth Factor Receptor 2 Antibody Suppresses Trastuzumab-Resistant Breast Cancer with Enhanced Nanoscale Binding
Source: ACS Nano. 2024 May 20;18(25):16126–40. doi: 10.1021/acsnano.4c00360 (PMC11210341; doi:10.1021/acsnano.4c00360)
Supplement: Supplementary file 1 — nn4c00360_si_001.pdf [file nn4c00360_si_001.pdf]

Supporting Information

Plant-Derived Anti-Human Epidermal Growth Factor Receptor 2 Antibody Suppresses Trastuzumab-Resistant Breast Cancer with Enhanced Nanoscale Binding

Chanyong Park[1]†, Kibum Kim[2], Yerin Kim[2], Rong Zhu[3], Lisa Hain[3], Hannah Seferovic[3], Min-Hyeok Kim[1], Hyun Joo Woo[4], Hyunju Hwang[2], Seung Ho Lee[4], Sangmin Kim[5], Jeong Eon Lee[6], Peter Hinterdorfer[3], Kisung Ko[2], Sungsu Park[1]*, Yoo Jin Oh[3]**

[1]School of Mechanical Engineering, Sungkyunkwan University, Suwon 16419, Korea

[2]Department of Medicine, Medical Research Institute, College of Medicine, Chung-Ang University, Seoul 06974, Korea

[3]Department of Applied Experimental Biophysics, Institute of Biophysics, Johannes Kepler University Linz, 4040 Linz, Austria

[4]Major of Nano-Bioengineering, College of Life Sciences and Bioengineering, Incheon National University, Incheon 22012, Korea

[5]Department of Breast Cancer Center, Samsung Medical Center, Sungkyunkwan University School of Medicine, Seoul 06351, Korea

[6]Division of Breast Surgery, Department of Surgery, Samsung Medical Center, Sungkyunkwan University School of Medicine, Seoul 06351, Korea

Corresponding authors: Kisung Ko: ksko@cau.ac.kr; Sungsu Park: nanopark@skku.edu; Yoo Jin Oh: yoo_jin.oh@jku.at

This PDF file includes: Figures S1 to S4

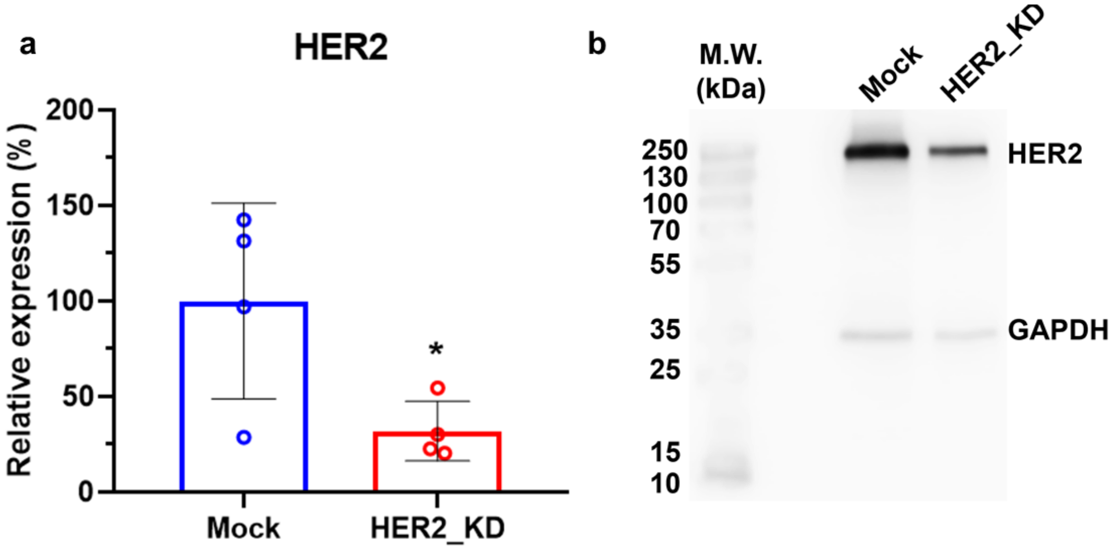

Figure S1. Confirmation of HER2 KD in BT474 cells. (a) Cells were subjected to treatment with siRNA targeting HER2 (HER2_KD) or control sequences (Mock). Subsequent analysis of HER2 gene expression post-siRNA knockdown was performed using quantitative reverse transcription PCR (qRT-PCR), with statistical significance determined by Student's t-test (***p<0.001, **p<0.01, *p<0.05). Glyceraldehyde 3-phosphate dehydrogenase (GAPDH) served as a reference for normalizing HER2 gene expression, and the expression levels in Mock-treated cells were utilized as the baseline for calculating the relative expression of the HER2 gene in HER2_KD cells.

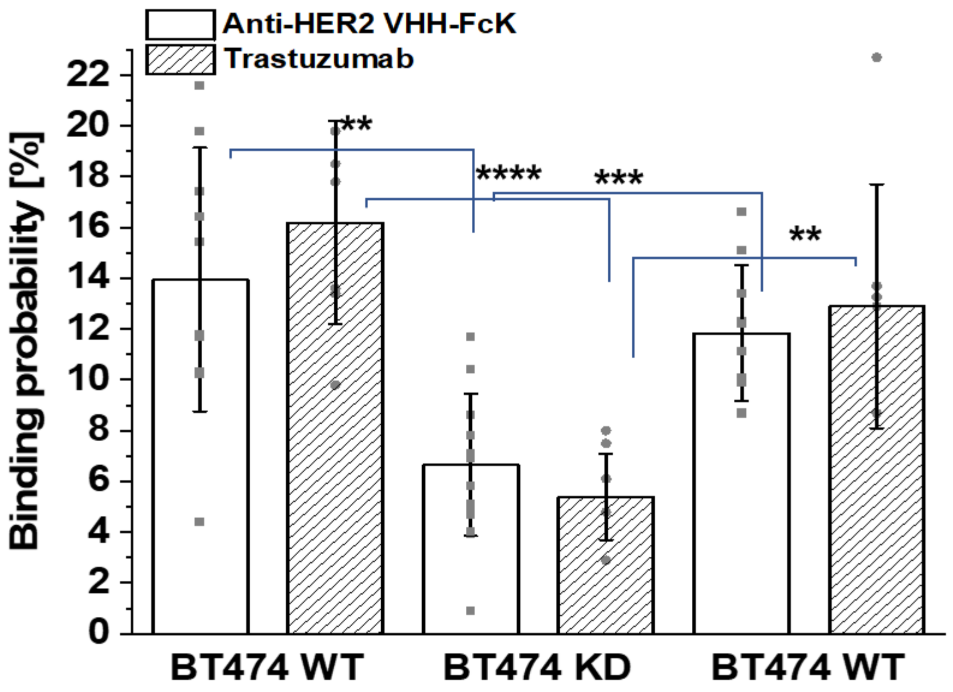

Figure S2. Single molecule force-spectroscopy control measurement. Binding probabilities of (a) anti-HER2 VHH-FcK and (b) Trastuzumab with BT474 WT, BT474 KD, BT474 WT (after KD cell measurement to confirm the functionality of measured tip) cells. Student's t-test; ****; $p<0.0001$, ***; $p<0.001$, **; $p<0.01$, *; $p<0.05$.

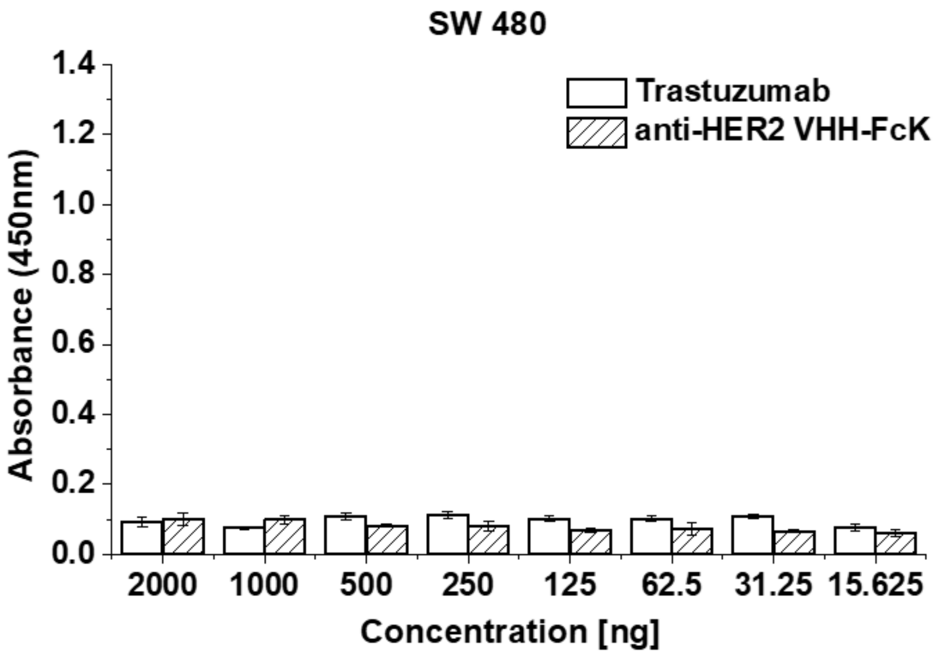

Figure S3. A cell ELISA to confirm binding activity of anti-HER2 VHH-FcK and Trastuzumab to SW480 colorectal cancer cells not expressing HER2 proteins (a negative control).

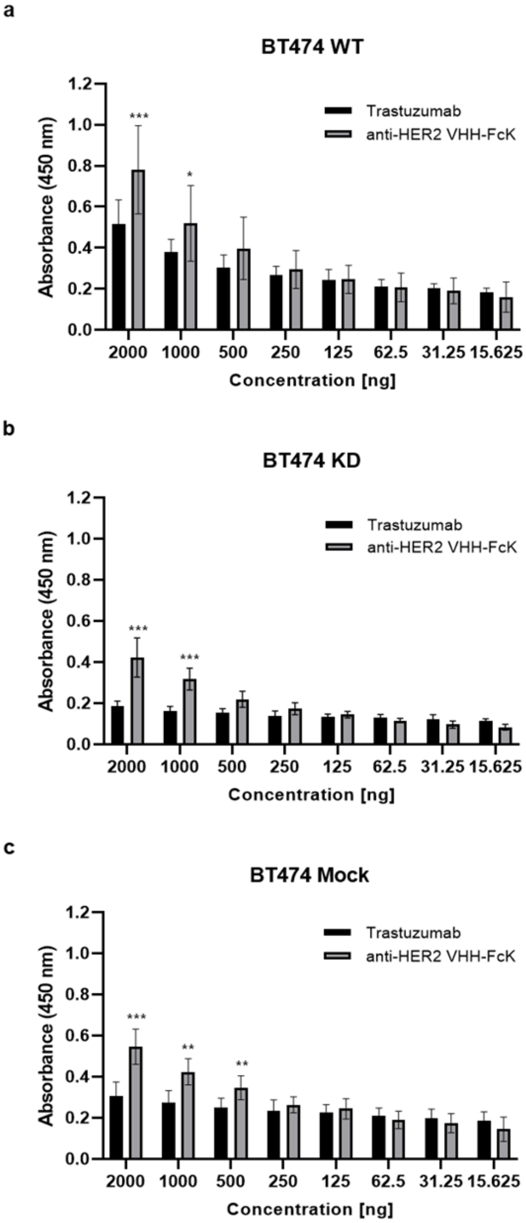

Figure S4. Cell ELISA to confirm binding activity of anti-HER2 VHH-FcK and Trastuzumab to BT474 WT, BT474 KD and BT474 Mock cells. HER2-positive BT-474 (BT474 WT) (a), HER2 knockdown BT-474 (BT474 KD) (b), and BT-474 cells transfected with the control RNA sequences (BT474 Mock) (c) were fixed on a 96-well cell culture plate using 4% paraformaldehyde (PFA) for 30 min at room temperature. Anti-HER2 VHH-FcK and Trastuzumab as a primary antibody were applied to BT474 WT, BT474 KD and BT474 Mock cells. Horse radish peroxidase (HRP)-conjugated goat anti-human IgG Fc antibody (1:8,000) was applied as a secondary antibody. Student's t-test; ***; $p<0.001$, **; $p<0.01$, *; $p<0.05$.
